# The Use of Platelet-Rich Plasma to Promote Cell Recruitment into Low-Molecular-Weight Fucoidan-Functionalized Poly(Ester-Urea-Urethane) Scaffolds for Soft-Tissue Engineering

**DOI:** 10.3390/polym11061016

**Published:** 2019-06-09

**Authors:** Géraldine Rohman, Credson Langueh, Salah Ramtani, Jean-Jacques Lataillade, Didier Lutomski, Karim Senni, Sylvie Changotade

**Affiliations:** 1 Tissue Engineering and Proteomics (TIP) team, CSPBAT UMR CNRS 7244, Université Paris 13, Sorbonne Paris Cité, 74 rue Marcel Cachin, 93000 Bobigny, France; credsonlangueh@gmail.com (C.L.); lutomski@univ-paris13.fr (D.L.); changotade@univ-paris13.fr (S.C.); 2 LBPS team, CSPBAT UMR CNRS 7244, Université Paris 13, Sorbonne Paris Cité, 99 avenue Jean-Baptiste Clément, 93430 Villetaneuse, France; ramtani@univ-paris13.fr; 3 Institut de Recherche Biomédicale des Armées, Unité de Thérapie Cellulaire et Réparation Tissulaire, Site du Centre de Transfusion Sanguine des Armées “Jean Julliard” de Clamart, BP 73, 91223 Brétigny-sur-Orge Cedex, France; jjlataillade@gmail.com; 4 Ecole de biologie Industrielle, 49 avenue des Genottes, 95885 Cergy Cedex, France; senni_k@yahoo.com

**Keywords:** fucoidan, PRP, poly(ester-urea-urethane), scaffold, tissue engineering

## Abstract

Due to their elastomeric behavior, polyurethane-based scaffolds can find various applications in soft-tissue engineering. However, their relatively inert surface has to be modified in order to improve cell colonization and control cell fate. The present study focuses on porous biodegradable scaffolds based on poly(ester-urea-urethane), functionalized concomitantly to the scaffold elaboration with low-molecular-weight (LMW) fucoidan; and their bio-activation with platelet rich plasma (PRP) formulations with the aim to promote cell response. The LMW fucoidan-functionalization was obtained in a very homogeneous way, and was stable after the scaffold sterilization and incubation in phosphate-buffered saline. Biomolecules from PRP readily penetrated into the functionalized scaffold, leading to a biological frame on the pore walls. Preliminary in vitro assays were assessed to demonstrate the improvement of scaffold behavior towards cell response. The scaffold bio-activation drastically improved cell migration. Moreover, cells interacted with all pore sides into the bio-activated scaffold forming cell bridges across pores. Our work brought out an easy and versatile way of developing functionalized and bio-activated elastomeric poly(ester-urea-urethane) scaffolds with a better cell response.

## 1. Introduction

Tissue engineering is a multi-step and multi-component approach, aiming to combine a porous and biodegradable polymeric structure with bioactive factors in order to elaborate appropriate scaffolds conducive to cell adhesion, proliferation and function maintenance, as well as vascularization and tissue regeneration [1]. As a consequence, compelling requirements have been identified for scaffold design: (1) Being easily shaped for large-scale manufacturing through a simple and controlled process, and allowing material morphologies that can fit in the defect geometry in order to correctly define and maintain the space for tissue regeneration; (2) possessing a multi-scale interconnected porous structure, enabling cell migration, provision of oxygen and nutrients to engineered tissue prior to vascularization, as well as the evacuation of metabolic waste; (3) surface properties that could support and enhance the delivery of bio-factors in order to stimulate cell proliferation, cell differentiation, matrix remodeling and angiogenesis. One target of tissue engineering is to ensure an effective colonization of scaffolds by cells, which can lead subsequently to tissue development. As a consequence, classical tissue engineering approaches rely on the seeding of cells onto the scaffold to potentiate the biomaterial regenerative properties. However, the seeding-time, the cost, the heterogeneity of the scaffold colonization, a loss of cell viability and proliferation are critical obstacles [2]. In contrast, cell-free based-tissue engineering is a straightforward approach leaning on inductive scaffolds capable of recruiting resident cells and guiding them to form a functional structure. Rapid cell infiltration, migration and proliferation into the porous biomaterial are of high importance for tissue regeneration, and may be enhanced by the delivery of biological cues from optimized scaffolds.

Various scaffolds, based on natural, synthetic and composite materials, have been proposed for tissue engineering and are described in the literature. In the field of soft-tissue engineering, the choice of the material used for the scaffolding matrix is limited by their mechanical stiffness, since most non-mineralized tissues to replace exhibit elastic moduli ranging from 0.01 to 1000 kPa [3]. As a consequence, elastomeric polyurethane-based scaffolds have found various applications in soft-tissue engineering, due to their good biocompatibility, rubber-like elasticity, high fatigue resistance, easiness of elaboration into different morphologies, and their versatility in terms of tailoring degradation rates [4,5]. However, polyurethane-based scaffolds exhibit an inert surface that has to be modified in order to improve cell colonization and to control cell fate. Therefore, a variety of approaches have been proposed in the literature to incorporate functional groups or immobilize biomolecules [5,6]. To improve scaffold efficiency and provide biological cues that promote tissue regeneration, it has been shown that the addition of growth factors presents great advantages [7]. Lately, platelet rich plasma (PRP) has been increasingly used in various tissue regeneration applications, since PRP contains more than 300 biologically-active molecules, with a high levels of growth factors, including the transforming growth factor β (TGF-β), insulin-like growth factor 1 (IGF-1), platelet-derived growth factor (PDGF), fibroblast growth factor (FGF). PRP also contains adhesion molecules, and is a vast reservoir of proteins [8,9,10]. PRP has been widely used as a therapeutic tool in the orthopedic field, because it allows the delivery of osteogenic and angiogenic factors [11,12]. The effect of PRP on various cell types has been studied in vitro, and PRP demonstrates adhesive, proliferative, differentiative and chemo-attractive properties [10,13,14]. For instance, PRP can stimulate dermal fibroblast migration and proliferation, showing its potential in wound treatment and skin rejuvenation [15]. Therefore, the incorporation of PRP in scaffolds is an advantageous approach, because it represents a simple, efficient and cost-effective method, allowing the immobilization of a number of highly-concentrated bioactive factors, creating an optimized micro-environment, and impacting upon tissue regeneration [10,16,17,18,19]. Various approaches have been developed to immobilize growth factors inside the scaffolds, and these may be envisioned for immobilizing PRP, such as physical encapsulation, absorption onto the biomaterial surface, layer-by-layer self-assembly, covalent conjunctions and extracellular matrix-based binding approaches [20]. In the latter bioaffinity-tethering approaches via charge interactions, sulfated glycosaminoglycans, such as heparin, have been widely used to design scaffolds with a highly spatio-temporal delivery capacity [21,22]. However, both polysaccharide extraction from animal tissue and chemical synthesis can be difficult, and can lead to macromolecules with various purity, structures, sulfation degrees and molecular weight dispersity. As a consequence, heparin-mimicking polymers which have been more structurally defined offer great advantages. In turn, the use of non-animal sulfated polysaccharides, such as fucoidans from brown seaweed, could be a clever approach. Attempts to immobilize fucoidan within polysaccharide scaffolds demonstrated that the content of fucoidan increased with its molecular weight. However, better growth factor retention was found for molecular weights in the range 20–40 kDa [23,24]. Indeed, low-molecular-weight (LMW) fucoidans are largely described as heparin mimetic, since they are able to protect and promote a number of growth factors, such as FGF-2 and vascular endothelial growth factor (VEGF), and to regulate tissue homeostasis [25,26,27,28,29,30]. 

LMW fucoidans have been investigated in therapeutic applications when used in soluble forms, as well as in medicine applications as a part of drug carriers, imaging agents, nano-systems for diagnostic and biomaterials for tissue regeneration [31,32]. Despite their bioactive properties, LMW fucoidans remain relatively unexploited, and very few studies reported their use to functionalize polymeric scaffolds.

Porous biodegradable scaffolds based on poly(ester-urea-urethane) (PEUU) were developed in our laboratory with various multi-scale and interconnected porosities. The scaffolds were appropriate for cell adhesion, allowed cell spreading over the pore walls, as well as a proliferation and osteogenic differentiation of stem cells seeded onto the scaffold [33,34]. Preliminary in vivo experiments dedicated to bone regeneration demonstrated the biocompatibility and the osteoconductive and osteoinductive properties of the PEUU scaffolds. Indeed, PEEU scaffolds were capable of recruiting resident osteoblastic progenitor cells, guiding cell migration and proliferation into the scaffold and inducing cell differentiation into mature osteoblasts [34]. With the aim of developing scaffolds for cell-free based-soft tissue engineering that can allow cell recruitment and scaffold colonization, keeping in mind that cell infiltration, migration, proliferation, as well as collagenous matrix making must be enhanced, the present study focuses on the PEUU scaffold bio-activation through a convenient surface modification, that can provide an appropriate surface chemistry for growth factors retention and extracellular matrix deposit. Herein, low-molecular-weight fucoidan functionalized-poly(ester-urea-urethane) scaffolds (PEUUF) were synthesized by incorporating LMW fucoidan concomitantly with the scaffold elaboration. Thereafter, PEUUF scaffolds were bio-activated through the adsorption of platelet rich plasma (PRP) formulations. Finally, preliminary in vitro assays were assessed to demonstrate the improvement of the scaffold behavior towards fibroblast response.

## 2. Materials and Methods

### 2.1. Materials

All solvents were purchased from Fisher, and were used as received. Phosphate buffered saline solution (PBS), Dulbecco’s modified Eagle’s medium (DMEM), fungizone antimycotic (Fz), penicillin (Pen), streptomycin (Strep) and trypsin-EDTA were supplied by Gibco Life Technologies (Dún Laoghaire, Ireland). Triol poly(ε-caprolactone) oligomers, hexamethylene diisocyanate (HMDI), Span^®^ 80, dibutyltin dilaurate (DBTDL), collagenase, paraformaldehyde (PFA), sodium chloride (NaCl), hydrochloric acid (HCl), picric acid, Sirius red, toluidine blue and hematein were purchased from Sigma-Aldrich, and Coomassie blue from BIO-RAD (Hercules, CA, USA). Low-molecular-weight (LMW) fucoidan (16–20 kDa, 40 wt% sulfate) was provided by the Solabia Group (Pantin, France). Fetal bovine serum (FBS) was obtained from PANBiotech GmbH (Aidenbach, Germany).

Heparin and clinical platelet rich plasma (PRP) formulations were supplied by the Centre de Transfusion Sanguine des Armées (CTSA) “Jean Julliard”—Hôpital d’Instruction des Armées Percy (Clamart, France). PRP was collected from platelet donations from healthy patients. Clinical PRP formulations were prepared from pools of platelets obtained from the CTSA Blood Bank.

Human dermal fibroblasts were isolated, using standard procedures, from the foreskins of 4-year-old children with the informed consent of their parents. Fibroblasts were used for the experiments at passage 7. The study was conducted in accordance with the Declaration of Helsinki and applicable local regulatory requirements and laws, that is to say the article L.1243-4 of the French Public Health Code. Given its special nature, surgical residue is subject to specific legislation included in the French Code of Public Health (anonymity, gratuity, sanitary/safety rules). This legislation does not require prior authorization by an ethics committee for any sampling or the use of surgical waste.

### 2.2. Elaboration of PEUUF Scaffolds

PEUUF scaffolds were obtained through the use of the high internal phase emulsion (HIPE) process by incorporating LMW fucoidan into the same high internal phase emulsion (HIPE) aqueous phase. An example of the preparation of a PEUUF scaffold is given hereafter. LMW fucoidan (0.34 g) was dissolved in 34 mL of sterile distilled water (0.2 μm filtered). Triol poly(ε-caprolactone) oligomers (1.3 g) and Span^®^ 80 (1.3 g) were placed in a reactor and dissolved in toluene (4.8 mL). Thereafter, the cross-linking agent HMDI (1.04 mL) and DBTDL catalyst (600 μL) were added under stirring using a mechanical stirrer. Finally, the aqueous fucoidan solution (C = 10 mg mL^−1^) was slowly introduced under stirring, leading to a stable paste-like emulsion. The HIPE was transferred in a mold and heated in an oven at 55 °C for 22 h followed by 2 h at 90 °C. Subsequently, the material was unmolded, and water and the excess of solvent were removed by squeezing. After 3 days of air-drying, the scaffold was cut into discs with a thickness of 2 mm and washed 2 times in dichloromethane for 24 h, then 24 h in dichloromethane/hexane 50/50 vol%, followed by 24 h in hexane and finally 2 times in distilled water for 24 h.

### 2.3. Bio-Activation of PEUUF Scaffolds with PRP Formulations

First, PEUUF scaffolds were immersed in sterile water for 1 h under a vacuum system, followed by a 4 h immersion after a water change. Thereafter, scaffolds were immersed for 1 h in 70 vol% ethanol under a vacuum system. Finally, scaffolds were rinsed in sterile water overnight and autoclaved in a wet condition. After sterilization, scaffolds were incubated at 37 °C in a humidified atmosphere of 5% CO_2_ in DMEM/Pen (100 IU.mL^−1^)/Strep (100 µg.mL^−1^)/Fz (2.5 µg.mL^−1^) for 12 h, and thereafter 16 h in DMEM/Pen (100 IU.mL^−1^)/Strep (100 µg.mL^−1^) supplemented with PRP (15 vol%). Finally, scaffolds were rinsed once in PBS.

### 2.4. Scaffold Characterization

Scaffolds were characterized by the determination of their density, porosity and pore interconnectivity (volumetric adsorption ratio), as previously described [33]. The scaffold morphology was monitored using an environmental scanning electron microscope (ESEM TM3000 - Hitachi, Tokyo, Japan), operating at an accelerated voltage of 15 kV, and a digital microscope Keyence VHX-5000 (Osaka, Japan). The scaffold chemical composition was monitored by Fourier-transformed infrared spectroscopy (FTIR Nicolet 380–Thermo Fisher Scientific, Waltham, MA, USA) in an attenuated total reflectance mode (ATR–Smart Omni Sampler, Thermo Fisher Scientific, Waltham, MA, USA) within the range 700–4000 cm^−1^ with a resolution of 4 cm^−1^. The scaffold effective modulus of elasticity E_1_* was determined during compression in the axial direction to the foam rise with a 500 N force range, 7 mm displacement range and 5 mm min^−1^ testing speed, as previously described [35]. The scaffold number average molecular weight between cross-links ${\bar{M}}_{c}$ was determined through the swelling measurements in toluene up to the swelling equilibrium, as previously described [35]. The scaffold hydrophilicity/hydrophobicity was determined through the water contact angle measurements using a Digidrop Model DS GBX apparatus (GBX Scientific, Tallaght, Ireland) and Windrop++ software, and through the water uptake. The scaffolds were weighed before water immersion (W_dry_) and after removing from distilled water (W_wet_). The water uptake was calculated using Equation (1):

Water Uptake = (W_wet_ ‒ W_dry_) × 100/W_dry_ (1)

The evidence of scaffold functionalization by LMW fucoidan was monitored by Fourier-transformed infrared (FTIR)-attenuated total reflection (ATR) spectroscopy, and the amount of fucoidan immobilized inside the scaffold was determined using a toluidine blue assay [36]. Ground scaffolds were immersed for 1 h in 2 mL of H_2_O/0.2 wt% NaCl solution and 1 mL of toluidine blue solution (C = 0.062 mg.mL^−1^ in 0.01M HCl/0.2wt% NaCl). Thereafter, 3 mL of hexane were added to the mixture and it was shaken well. The aqueous layer was then extracted and the absorbance measured at 632 nm with a UV-visible spectrophotometer (UVM 340 spectrophotometer, Mettler Toledo, Columbus, OH, USA). 

A linear relationship between the absorbance of residual toluidine blue in the aqueous phase and the concentration of fucoidan in aqueous solution was obtained from a calibration curve and used to determine the amount of fucoidan immobilized inside the scaffold.

The evidence of PRP immobilization was monitored by FTIR-ATR spectroscopy and by imaging the scaffold stained by Coomassie blue with a digital microscope Keyence VHX-5000. The amount of PRP immobilized inside the scaffold was determined using a 2D Quant kit (GE Healthcare, Chicago, IL, USA).

### 2.5. Evaluation of Scaffold Stability

The scaffold stability was assessed through degradation tests in accordance with ISO 10993-13 guidelines. After sterilization by autoclaving, 0.1 g of PEUUF scaffolds were immersed in 1 mL of degradation medium composed of DMEM/Pen (100 IU.mL^−1^)/Strep (100 µg.mL^−1^)/Fz (2.5 µg.mL^−1^) and incubated at 37 °C or 90 °C for different periods of time. The pH of the degradation medium was monitored during the course of the study. At the end of each time point, the scaffolds were removed from the medium and washed extensively in distilled water. Subsequently, these scaffolds were air-dried up to a constant mass, and then they were characterized by FTIR-ATR analyses, ESEM imaging, as well as through the determination of the water uptake, water contact angle, number average molecular weight between cross-links ${\bar{M}}_{c}$, and mass loss from the scaffold initial mass and their residual mass after drying.

### 2.6. Scaffold Colonization by Fibroblasts

Fibroblasts at passage 7 resuspended in DMEM/Pen (100 IU.mL^−1^)/Strep (100 µg.mL^−1^) supplemented with FBS (10%) were seeded onto 24-well plates at 4 × 10^4^ cells per well. Fibroblasts were cultured at 37 °C in a humidified atmosphere of 5% CO_2_ until an apparent 80% confluence was reached. Thereafter, the medium was removed, and one scaffold (1 cm in diameter, 2 mm in height) per well was carefully set down over the 80% confluent cell layer and incubated in DMEM/Pen (100 IU.mL^−1^)/Strep (100 µg.mL^−1^) supplemented with PRP (5 vol%) and heparin (2 IU.mL^−1^) at 37 °C in a humidified atmosphere of 5% CO_2_ for up to 35 days. The medium was changed once a week. The scaffolds were tested in triplicate in all experiments.

At 10, 25 and 35 days, the scaffolds were carefully rinsed with PBS and incubated at 37 °C for 10 min in a solution of trypsin (0.05%) and collagenase (0.025%). Thereafter, the supernatant was recovered and centrifuged, and the pellet of cells was resuspended. In order to take off all the cells from the scaffold, this operation is repeated three times. Then, the total number of cells was determined by counting with a Coulter Counter Z1 (Beckman Coulter, Brea, CA, USA) to assess the efficiency of cell migration into the scaffolds.

For ESEM imaging, the scaffolds were rinsed in PBS, fixed in PFA (4%) and stored in ethanol 70 vol% up to observation. Images were carried out using a Hitachi TM3000 ESEM operating at 5 kV and equipped with a Peltier stage operating at −4 °C. For staining, scaffolds were rinsed in PBS, fixed in PFA (4%) for 24 h at 4 °C, and rinsed in distilled water. For Sirius Red staining, scaffolds were immersed in saturated picric acid with 0.1% Sirius Red for 30 min and rinsed in distilled water [37]. For cell staining, scaffolds were immersed in hemalun solution (0.2% of hematein in 5% aqueous potassium alum) for 5 m and rinsed in tap water for 5 min [38]. Finally, the stained scaffolds were stored in ethanol 70 vol% up to observation. The scaffold staining was monitored using an Alicona InfiniteFocus microscope (Alicona Imaging GmbH, Raaba/Graz, Austria).

### 2.7. Data Analysis

For the measured parameters of the scaffolds and the in vitro studies, the values are expressed as mean ± standard deviation (SD). Statistical analysis was performed using the Statistical Package for the Social Sciences (SPSS) software. For every population, group normality was checked by the Kolmogorov-Smirnov’s test. The homogeneity of variances between the groups was assessed by a Levene’s test. 

Kruskal-Wallis or Welch’s tests were performed for the inspection of any statistical differences between the means. In all statistical evaluations, p < 0.05 was considered statistically significant.

## 3. Results

### 3.1. PEUUF Scaffolds

**Figure 1:**
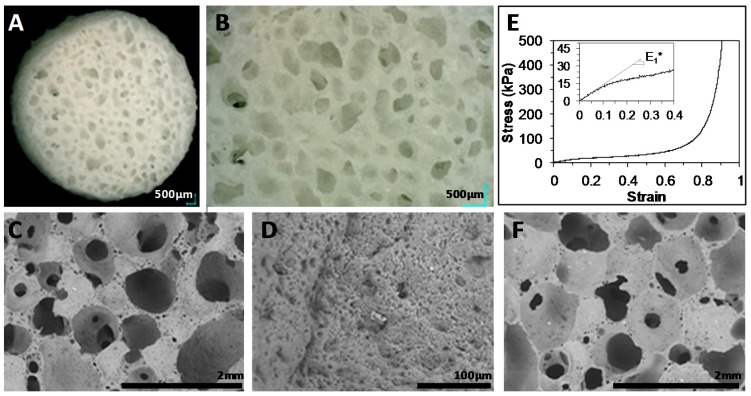
(**A**,**B**): Digital microscope images of functionalized-poly(ester-urea-urethane) scaffolds (PEUUF) scaffolds. (**C**,**D**): Environmental scanning electron microscope (ESEM) images of PEUUF scaffolds. **E**: Mechanical behavior of PEUUF scaffolds under stress modes of compression. **F**: ESEM image of PEUUF scaffolds after 7 months of incubation at 37 °C in the degradation medium. The low-molecular-weight (LMW) fucoidan concentration in the aqueous phase (34 mL) of the High Internal Phase Emulsion (HIPE) used for functionalization = 10 mg mL^−1^).

The PEUUF scaffolds were synthesized in a one-step reaction through the cross-linking of a high internal phase emulsion (HIPE) that contained LMW fucoidan in its aqueous phase. When prepared with 34 mL of a 10 mg mL^−1^ fucoidan solution, the resulted emulsion had a stable paste-like texture with no phase separation. After the removal of the aqueous internal phase and the washing of the cross-linked scaffolds, foams were obtained with highly interconnected pores without closed voids (Figure 1A,C). Indeed, the pore interconnectivity was found to be 95.1 ± 5.3%, indicating that the pores were all very well interconnected. The foams exhibited a high porosity of 83.4 ± 3.1% and a multi-scale pore size ranging from 30 to 2000 μm with an average pore size of approximately 500 μm (Figure 1B,C). Moreover, the surface of the pore walls exhibited a fine rugosity (Figure 1D). The mechanical behavior of the PEUUF scaffolds was determined through compression test (Figure 1E). The compressive stress-strain response of the scaffolds was typical of an elastomeric open-cell foam with three domains: A visco-elastic domain characterized by a linear stress*-*strain relationship, a post-buckling domain characterized by a long elastic plateau due to pore collapse, a post-complete densification domain where the strain increases rapidly as all the pores have collapsed [39]. The curve slope in the visco-elastic domain allowed the determination of the effective elasticity modulus E_1_* of the PEUUF scaffold, which was found to be 103 ± 23 kPa.

**Figure 2:**
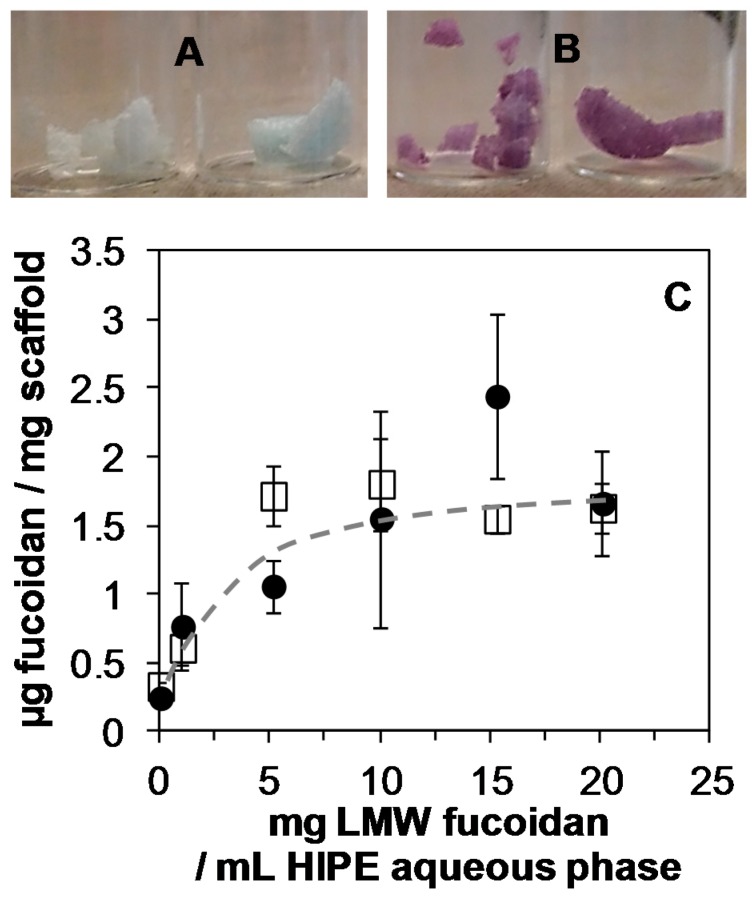
A,B: Scaffold staining (after washing) due to complexation of toluidine blue with LMW fucoidan for the fucoidan concentration in the HIPE aqueous phase (34 mL) of 0 mg mL^−1^ (**A**) and 10 mg mL^−1^ (**B**). **C**: Amount of LMW fucoidan immobilized inside scaffolds before (☐) and after washing (●) as a function of the LMW fucoidan concentration in the HIPE aqueous phase.

The LMW fucoidan immobilization inside the scaffold was obtained in a very homogeneous way throughout the scaffold thickness (Figure 2B), and it was clear that the complexation of toluidine blue onto the PEUUF scaffold was only due to the presence of LMW fucoidan, as a scaffold prepared without fucoidan in the emulsion remained nearly colorless (Figure 2A). The fucoidan immobilization inside the scaffold increased as the concentration of fucoidan in the aqueous phase of the HIPE increased up to 10 mg mL^−1^ (Figure 2C). 

Higher fucoidan concentration in the HIPE did not lead to an increase in the fucoidan immobilization, which reached around 1.6 μg per mg of scaffold. It was also found that varying the aqueous phase content in the HIPE impacted the amount of immobilized fucoidan. Indeed, 1.27 ± 0.1 and 2.16 ± 0.3 μg of fucoidan per mg of scaffold were measured when the HIPE was prepared, respectively, with 20 mL and 50 mL of a 10 mg mL^−1^ fucoidan aqueous solution. However, the modification of the aqueous phase content in the HIPE impacted the pore sizes. As a consequence, a concentration of fucoidan of 10 mg mL^−1^ and an aqueous phase content of 34 mL in the HIPE were chosen for the bio-activation study. Finally, the fucoidan immobilization was stable after the scaffold washing (Figure 2C).

**Figure 3:**
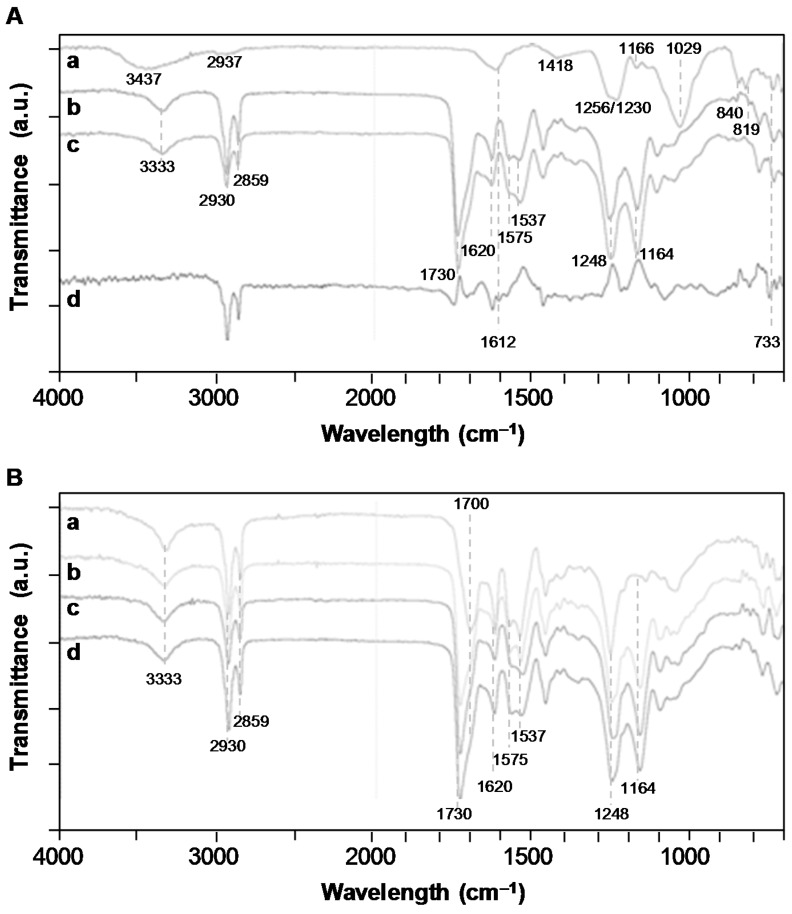
**A**: Fourier-transformed infrared (FTIR)-attenuated total reflection (ATR) spectroscopy spectra of a/LMW fucoidan; b/PEUUF scaffold; c/Non-functionalized scaffold synthesized in absence of fucoidan; d/Subtraction spectrum of functionalized and non-functionalized scaffolds. **B**: FTIR-ATR spectra of a/PEUUF scaffold after 70 days of incubation at 90 °C in the degradation medium; b/PEUUF scaffold after 11 days of incubation at 90 °C in the degradation medium; c/PEUUF scaffold after 7 months of incubation at 37 °C in the degradation medium; d/PEUUF scaffold before incubation in the degradation medium. (The LMW fucoidan concentration in the aqueous phase of the HIPE used for functionalization = 10 mg mL^−1^).

The fucoidan immobilization was also put in evidence through FTIR-ATR analysis (Figure 3A). The spectrum of the PEUUF scaffold presented the significant bands of poly(ester-urea-urethane)-based materials, such as urethane hydrogen-bonded –NH stretching at 3333 cm^−1^, –C=O groups for urethane and ester at 1730 cm^−1^ and for urea at 1620 cm^−1^, urea –CNH groups at 1575 cm^−1^ and urethane –NH bending at 1537 cm^−1^, –CN stretching and –NH bending associated with urethane groups at 1248 cm^−1^, and finally the stretching vibrations of the ester groups at 1164 cm^−1^ [33]. In addition, the PEUUF scaffold exhibited two bands at 840 cm^−1^ and 819 cm^−1^ associated with a C–O–S stretching of the fucoidan sulfate groups [40,41]. The subtracted spectrum also put in evidence two more bands typical for polysaccharides at 1612 cm^−1^ due to the in-plane ring CCH, OCH and COH vibrations and at 733 cm^−1^ for the C–O–S stretching of the sulfate groups [42,43]. Finally, the presence of fucoidan inside the scaffold modified the surface wettability, since the water contact angle of PEUUF scaffolds was found to be 79 ± 4° by comparison with 96 ± 10° for any scaffolds synthesized in the absence of fucoidan.

**Figure 4:**
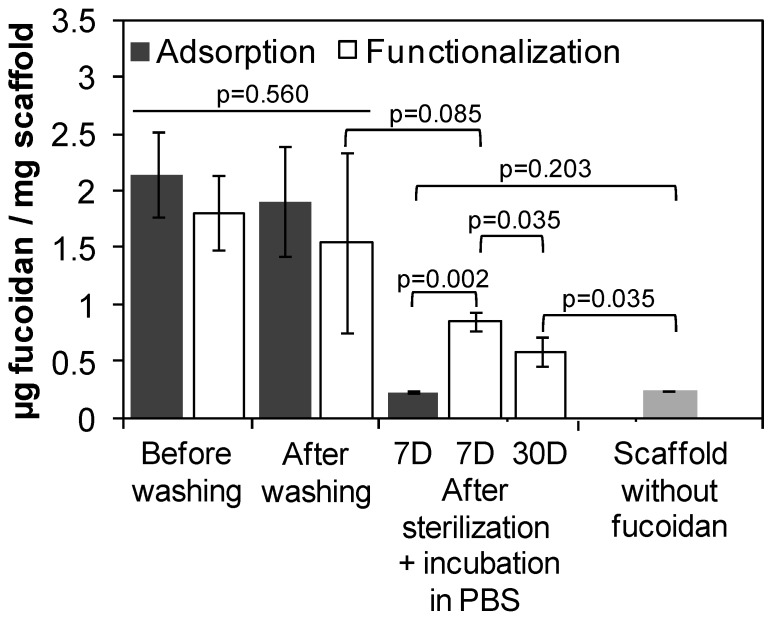
Amount of LMW fucoidan immobilized inside scaffolds when adsorbed after the scaffold elaboration (adsorption), or when directly incorporated during the scaffold elaboration (functionalization) before washing, after washing and after incubation in PBS by comparison with scaffolds synthesized in the absence of fucoidan. (The LMW fucoidan concentration in the aqueous phase used for adsorption or functionalization = 10 mg mL^−1^).

The stability of the functionalization was studied by quantifying the amount of fucoidan remaining inside the scaffold after washing, sterilization and incubation in PBS during 7 days and 30 days (Figure 4). There was no statistically significant difference in the amount of fucoidan between the adsorption and functionalization methods either before or after washing (p = 0.560). No fucoidan remained inside the scaffold after sterilization and 7 days of incubation in PBS when fucoidan was only adsorbed after the scaffold elaboration (p = 0.203 between incubated scaffolds containing adsorbed fucoidan and scaffolds synthesized in an absence of fucoidan). Notwithstanding, the functionalization developed in this study was stable, since no statistically significant difference in the amount of immobilized fucoidan was found after the washing of PEUUF scaffolds and after sterilization and 7 days of incubation in PBS (p = 0.085). After 30 days of incubation in PBS, the amount of immobilized fucoidan slightly decreased (p = 0.035), but was still statistically significantly higher than scaffolds synthesized in the absence of fucoidan (p = 0.035) and scaffolds containing adsorbed fucoidan after sterilization and 7 days of incubation in PBS (p = 0.031).

**Figure 5:**
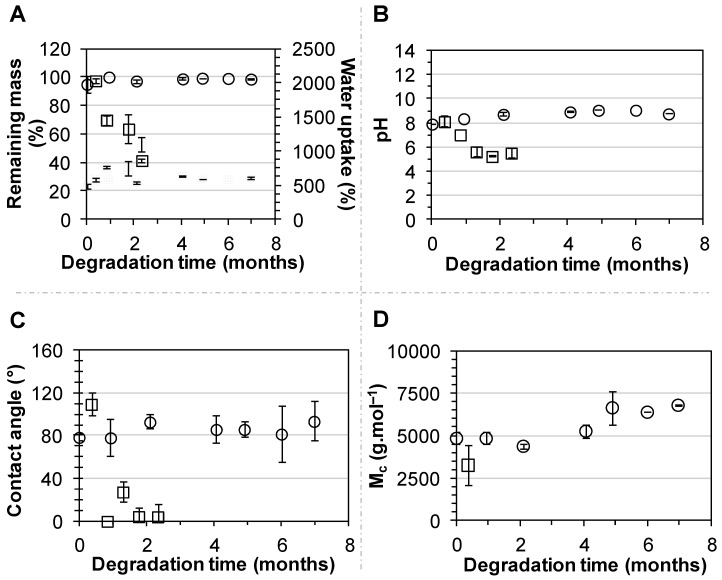
Incubation of PEUUF scaffolds in the degradation medium at 37 °C (○) and 90 °C (☐) over a period of 7 months and 70 days, respectively: (**A**) Scaffold remaining mass (open symbols) and water uptake (full symbols); (**B**) Medium pH; (**C**) Water contact angle; (**D**) Scaffold number average molecular weight between cross-links ${\bar{M}}_{c}$. (The LMW fucoidan concentration in the aqueous phase of the HIPE used for functionalization = 10 mg mL^−1^).

The stability of PEUUF scaffolds was studied for up to 7 months in the DMEM medium at 37 °C. No variation in scaffold mass, water uptake and water contact angle was noticed (Figure 5A,C). No release of acidic degradation products was evidenced, since there was no variation of the medium pH (Figure 5B). However after 7 months of incubation, a very small variation of the PEUUF composition was detected by an FTIR-ATR analysis through a decrease of 2.8% of the intensity of the peak at 1164 cm^−1^, corresponding to the stretching vibrations of the ester groups (Figure 3B). An increase in the number average molecular weight between cross-links ${\bar{M}}_{c}$ due to chain scissions was also noticed (Figure 5D). Moreover, the porosity slightly decreased after 7 months of incubation (78.4 ± 1.1%), while no significant variation of the porous morphology of the PEUUF scaffold was evidenced (Figure 1F). Finally, an accelerated aging was also performed at 90 °C. At this degradation temperature, it was noticed that mass loss started to occur after 11 days of incubation (Figure 5A) that led to a decrease or the impossibility to measure the number average molecular weight between cross-links ${\bar{M}}_{c}$ (Figure 5D), because the scaffold became very brittle. An increase of the water uptake (Figure 5A) and a drastic decrease of the water contact angle (Figure 5C) were also noticed, which was in accordance with the FTIR-ATR analysis (Figure 3B). Indeed, the peak at 1164 cm^−1^ of the ester groups decreased by 11.3% after 11 days of incubation in the degradation medium, and up to 71.8% after 70 days. Acidic degradation products were released during the degradation, as attested by the variation of the medium pH (Figure 5B). Overall, it was possible to conclude that the PEUUF scaffold life-time at 90 °C was approximately 11 days. The scaffold life-time at 37 °C was estimated to be that of the scaffold life-time at 90 °C multiplied by the factor *f* ranging from 39.4 to 128.6 as calculated by Equation (2) [44]:

(2) $$f=Q_{10}^{\Delta T/10}$$

In Equation (2), ΔT is the difference between the elevated temperature used to accelerate the degradation process (90 °C), and the temperature at which to study the effects of degradation (37 °C); the aging factor Q_10_ was taken as 2–2.5 in accordance with the ASTM F1980-16 standard. Therefore, the PEUUF scaffold life-time at 37 °C was estimated to range between 14.2 and 46.5 months.

### 3.2. PEUUF Scaffold Bio-Activation with PRP

**Figure 6:**
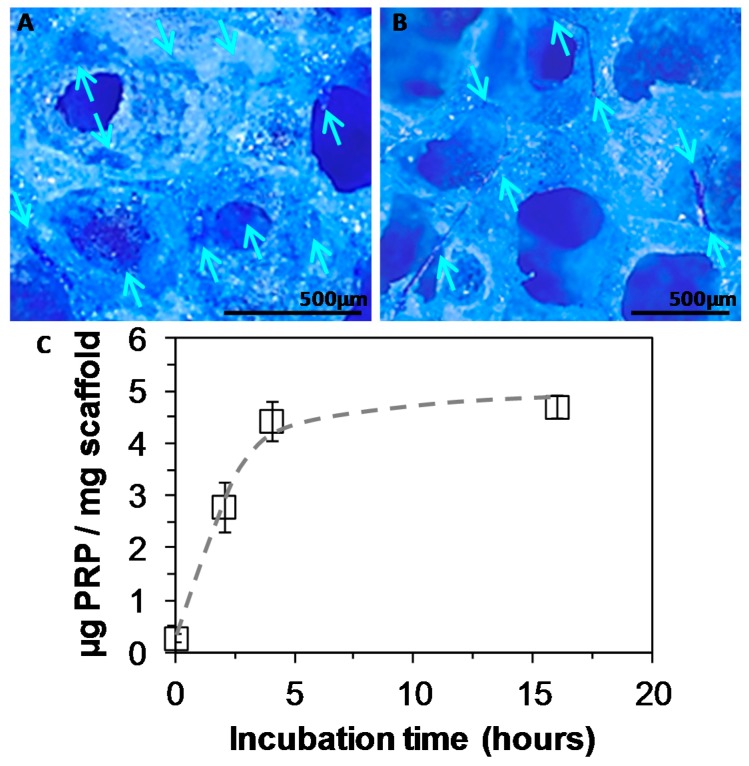
**A**,**B**: Staining of platelet rich plasma (PRP) adsorbed into PEUUF scaffolds due to complexation with Coomassie blue (PRP concentration used for bio-activation = 15 vol% (16 h of incubation)) (Arrows indicate some locations of PRP in images). **C**: Amount of PRP adsorbed into PEUUF scaffolds as a function of incubation time in a 15 vol% PRP solution (The LMW fucoidan concentration in the aqueous phase of the HIPE used for functionalization = 10 mg mL^−1^).

The scaffold bio-activation with PRP was carried out by impregnation after the sterilization of the PEUUF scaffolds. Biomolecules from PRP readily penetrated into the scaffold, and the density of the PRP adsorption depended on the scaffold immersion time in the PRP solution, as well as on the solution concentration. When incubated in a 15 vol% PRP solution, the scaffold bio-activation increased as the incubation time increased up to nearly 5 h (Figure 6C). A higher incubation time did not lead to an increase in the PRP adsorption, which reached around 4.7 μg per mg of scaffold. Moreover by this adsorption method, a biological frame covering the pore walls was obtained after 16 h of incubation in all tested PRP concentrations (10, 15 and 20 vol%) (Figure 6A). In addition, for a PRP concentration of 15 vol%, a fibrillar network appeared across pores while maintaining the open porosity of the scaffold (Figure 6B). When increasing the PRP concentration to 20 vol%, a thick hydrogel surrounded the scaffold and led to pore closure. As a consequence, the concentration of PRP used for the bio-activation was chosen to be 15 vol% for the in vitro assays.

**Figure 7:**
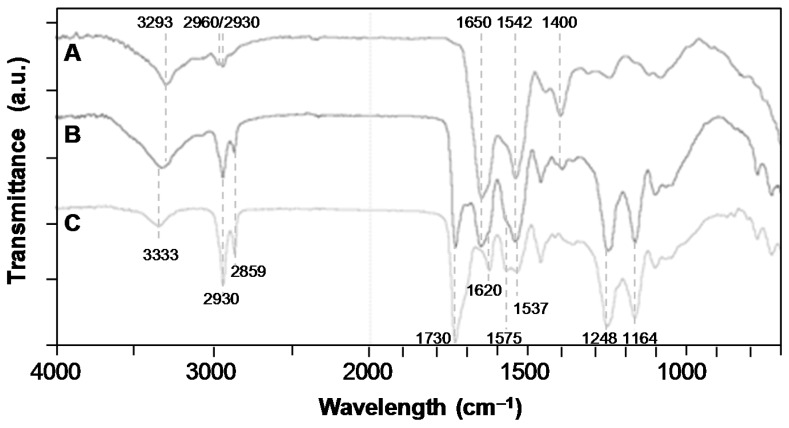
FTIR-ATR spectra of: A/Pure PRP (dry); B/PRP-PEUUF scaffold (dry); C/PEUUF scaffold (the LMW fucoidan concentration in the aqueous phase of the HIPE used for functionalization = 10 mg mL^−1^; and the PRP concentration used for bio-activation = 15 vol% (16 h of incubation)).

The PRP adsorption was also put in evidence through an FTIR-ATR analysis and by contact angle and water uptake measurements. The presence of PRP increased the hydrophilicity of the scaffold, since the water contact angle decreased from 79 ± 4° for PEUUF scaffolds to 32 ± 44° for PRP bio-activated ones (PRP-PEUUF). It has to be pointed out that the water drop spread slowly on PRP-PEUUF scaffolds. Moreover, the water uptake was determined by immersing dry scaffolds for 24 h in distilled water. It was found that the water uptake was enhanced from 277 ± 22% for the PEUUF scaffold to 367 ± 5% for the PRP-PEUUF ones. The FTIR-ATR analysis of those PRP-PEUUF scaffolds presented the significant bands of PEUUF, and also exhibited bands from proteins present in PRP, such as the amide I and II groups of the peptide bond at respectively, 1650 cm^−1^ and 1542 cm^−1^, the bending of CH_2/3_ groups present in amino acid side chains at 1400 cm^−1^, as well as the broadening of the band centered at 3293 cm^−1^ assigned to the protein NH groups (Figure 7) [45].

### 3.3. Cell Responses towards PEUUF and PRP-PEUUF Scaffolds

**Figure 8:**
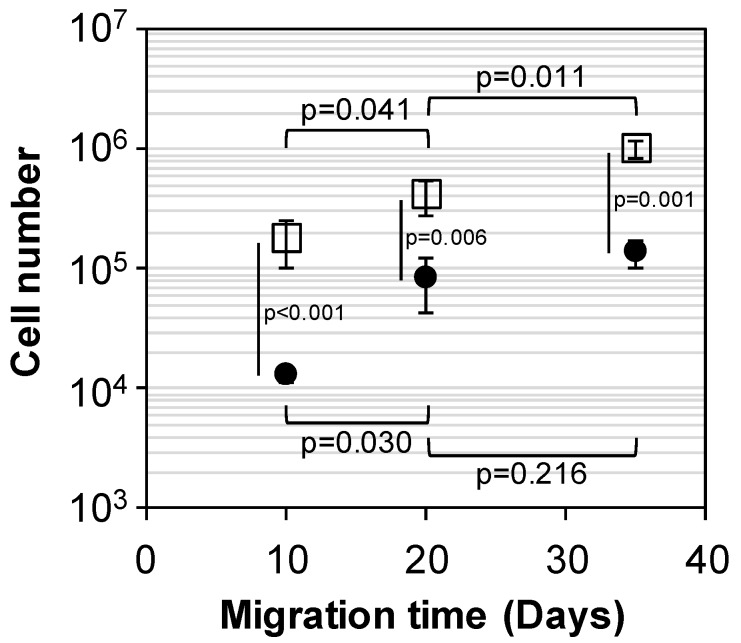
Cell number after 10, 20 and 35 days of fibroblast migration into PEUUF scaffolds (●) and PRP-PEUUF scaffolds (☐) (the LMW fucoidan concentration in the aqueous phase of the HIPE used for functionalization = 10 mg mL^−1^; the PRP concentration used for bio-activation = 15 vol% (16 h of incubation)).

The capability of scaffolds to recruit cells was carried out through migration assays by setting down scaffolds over a confluent cell layer. It was noticed that fibroblasts were able to migrate into the porous structure of both PEUUF and PRP-PEUUF scaffolds (Figure 8). However, the bio-activation with PRP drastically improved the cell recruitment and migration, since more cells were found in PRP-PEUUF scaffolds, as attested by the statistically significant difference of the cell number at each day of migration (p < 0.001 at 10 days, p = 0.006 at 20 days and p = 0.001 at 35 days). Moreover, the cell number increased continuously with migration time into the PRP-PEUUF scaffolds, while there was no statistically significant difference for the PEUUF scaffolds between 20 and 35 days of migration (p = 0.216).

**Figure 9:**
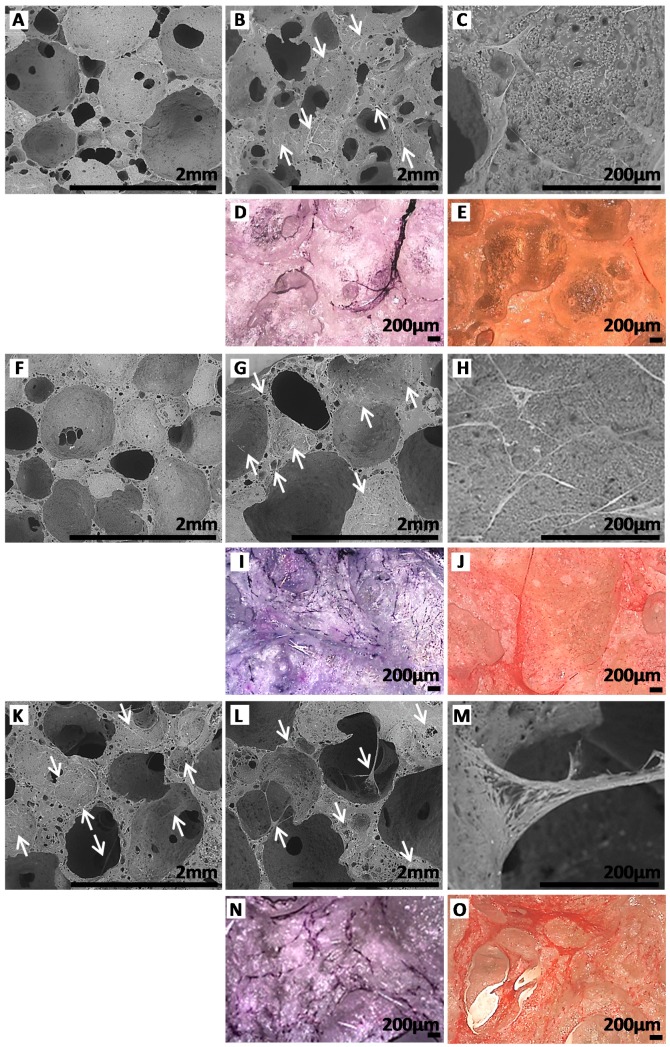
ESEM images of scaffolds after 10 (**A**,**F**,**K**) and 35 (**B**,**C**,**G**,**H**,**L**,M) days of fibroblast migration (Arrows indicate some locations of cells in ESEM images). Images after 35 days of fibroblast migration of scaffolds stained with Hemalun (**D**,**I**,**N**) and Sirius red (**E**,**J**,**O**). **A**,**B**,**C**,**D**,**E**: Non-functionalized scaffolds synthesized in absence of fucoidan and impregnated with PRP. **F**,**G**,**H**,**I**,**J**: PEUUF scaffolds. **K**,**L**,**M**,**N**,**O**: PRP-PEUUF scaffolds. (The LMW fucoidan concentration in the aqueous phase of the HIPE used for functionalization = 10 mg mL^−1^; while the PRP concentration used for bio-activation = 15 vol% (16 h of incubation)).

For PEUUF scaffolds, few cells were found within the scaffold after 10 days of migration (Figure 9F); and after 35 days, fibroblasts mostly attached to the surface of the pore walls (Figure 9G,I) and spread over them in an elongated pattern (Figure 9H). As expected from the cell number results, more fibroblasts were found inside the PRP-PEUUF scaffold from 10 days of migration (Figure 9K,L,N). Moreover, cells both attached and spread over the pore wall surface and interacted with all pore sides, leading to the bridging of cell layers across pores (Figure 9L,M). After 35 days of migration, the fibroblasts in the PRP-bio-activated and LMW-functionalized scaffold exhibited a higher staining of the collagenous matrix arranged in a more homogeneous network (Figure 9J,O). Furthermore, it has to be pointed out that, if cells were able to infiltrate and to attach on the pore walls of non-functionalized scaffolds synthesized in the absence of fucoidan and impregnated with PRP (Figure 9A,C,D), the 3D arrangement of these cells inside the scaffold porous structure and the increase of the collagen deposit were not found (Figure 9B,E), attesting the synergic effect of the scaffold functionalization by the LMW fucoidan and its bio-activation by PRP on the cell response. 

## 4. Discussion

To overcome the limitations of classical tissue engineering based on cell seeding onto scaffolds, cell-free approaches have been investigated, and the use of a cell-free scaffold as an off-the-shelf product presents a more reliable and foreseeable clinical solution [2,46,47,48,49]. Nevertheless, the use of cell-free scaffolds relies on the elaboration of a biomaterial capable of recruiting and guiding resident cells to stimulate tissue development. Since the interactions between a scaffold and the cells are mediated by the biomaterial surface energy, surface topography, surface functionality and surface stiffness, tissue regeneration requires the optimal combination of an optimized scaffold and biological cues [50]. The purpose of this study was to investigate the design, through a straightforward manufacturing and shaping method, of an elastomeric scaffold suitable for soft-tissue engineering, with an appropriate surface chemistry allowing the retention of biomolecules that can direct cellular processes. After the elaboration of PEUUF scaffolds, biological cues were introduced through bio-activation by PRP formulations.

### 4.1. PEUUF Scaffolds as Biomaterials for Soft-Tissue Engineering

Fucoidan is a natural anionic sulfated polysaccharide extracted from brown Algae that has been extensively studied in recent years, due to its numerous biological properties, such as anti-tumor, anti-coagulant, anti-thrombotic, anti-inflammatory and anti-oxidant effects [28,32,51,52]. As a matter of fact, the properties of these polysaccharides depend on their electrical charges, sulfation degree and molecular weight [25,53,54]. It has been demonstrated that low-molecular-weight fucoidans exhibit therapeutic potential in tissue engineering because fucoidans can influence cell adhesion, migration, proliferation and differentiation [25,52,55]. Indeed, fucoidans are able to bind growth factors providing an appropriate environment for cell migration and proliferation; and to regulate their bioavailability, which is of great interest for tissue engineering applications [56,57,58,59]. For instance, LMW fucoidans enhance FGF-2 activity, mobilize SDF-1 and promote in vivo angiogenesis [25]. Interestingly, LMW fucoidans also allow the protection of extracellular matrix and growth factors against proteolysis [27,60].

In our study, LMW fucoidan was easily immobilized during the scaffold elaboration using the HIPE method. HIPEs are characterized by a network of the droplet interfaces, the properties of which will determine the properties of the resulting scaffold [61]. As a consequence, the HIPE water-oil interface might provide opportunities to tune scaffold properties. Indeed, the poly(ester-urea-urethane) matrix was synthesized by a one-step polycondensation reaction between the isocyanate groups of the cross-linker and the alcohol groups of the caprolactone oligomers, which were contained in the organic continuous phase of the water-in-oil HIPE. During the scaffold elaboration, the LMW fucoidan, which was introduced in the aqueous phase, might readily be entrapped in the poly(ester-urea-urethane) matrix, or react via its hydroxyl groups with the isocyanate moities incorporated in excess in the emulsion. Since the fucoidan was in the aqueous phase, only a small amount can be incorporated at the HIPE water-oil interface. This point was attested by the increase of fucoidan immobilization in PEUUF scaffolds with the amount of aqueous phase in the HIPE formulation. The advantage of HIPE for scaffold fabrication is the control over porosity and pore size, as well as the interconnectivity through the modification of the internal phase volume fraction [33]. As a consequence for our study, the amount of the fucoidan solution (84 vol%) incorporated in the HIPE was dictated by the targeted scaffold morphology. The fucoidan molecular weight might also have an impact on the fucoidan immobilization. For instance, it was demonstrated that when polysaccharide electrospun fibers were elaborated in the presence of fucoidan, the content of immobilized fucoidan increased with its molecular weight [23,24]. Nevertheless, better growth factor retention was found for molecular weight in the range 20–40 kDa. Moreover, it has to be point out that HIPE relies on the droplet deformation by surface droplet stretching and the formation of plateau borders [61]. 

To provide stability, HIPE interface strength can be increased by using interfacial proteins. However, an enhanced rigidity of the droplets may prevent droplet deformation [62]. With the aim of developing a scaffold possessing a highly optimal porous structure appropriate for tissue development, and containing chemical groups that could retain biomolecules through ionic interactions, and potentially protect them against proteolytic degradation, PEUUF scaffolds were designed, favoring the use of more flexible LMW fucoidan with molecular weight in the range 16–20 kDa, and a high sulfation degree (40 wt% sulfate). By this elaboration method, the scaffold functionalization was obtained homogeneously in a very versatile and stable way. The amount of immobilized fucoidan, around 1.6 μg per mg of scaffold, was in the range of similar results reported from the literature for heparin and heparin-mimicking polymer functionalized scaffolds that exhibited growth factor retention properties and cell response modulating effects [23,24,36,63].

After scaffold elaboration, one critical task in the tissue engineering field is to find an appropriate sterilization process capable of achieving effective sterilization without leading to adverse post-sterilization effects. Steam sterilization is one of the most extensively used methods for biomaterial sterilization because it is effective, fast and simple. However, it can affect the strength, molecular weight and structural properties of biodegradable scaffolds [64]. In a previous study, we developed a method in order to steam sterilize under wet conditions poly(ester-urea-urethane)-based scaffolds without high adverse post-sterilization effects [34]. Here again, the micro-structural properties of PEUUF scaffolds was not affected by the sterilization process. Moreover, the fucoidan functionalization was stable after sterilization, and fucoidan was still present within the scaffold after 30 days of incubation in PBS. Concerning the in vitro scaffold stability at 37 °C, PEUUF scaffolds were quite stable for up to 7 months of incubation in the degradation medium. To estimate the over long timescale of scaffold degradation, accelerated aging was performed at 90 °C, and allowed to estimate the PEUUF scaffold life-time at 37 °C in the range of 14.2–46.5 months. The enhanced wettability of PEUUF scaffolds, due to the fucoidan immobilization, increased the degradation rate by comparison with non-functionalized scaffolds which exhibited a life-time at 37 °C in the range of 19.4–63.4 months [34]. It is well known that scaffold life-time is reduced in vivo because of more severe conditions. However, PEUUF scaffolds seem to be sufficiently stable for their used in soft-tissue engineering applications [65]. Obviously, the long term in vivo scaffold stability has to be confirmed by deep investigations, in particular to ascertain that the PEUUF scaffold degradation does not lead to a stimulation of inflammatory or immune systems.

### 4.2. PEUUF Scaffolds Allow Cell infiltration

The interactions between a scaffold and cells are mediated by the biomaterial surface energy, surface topography, surface functionality and surface stiffness [50]. As a consequence, the structural and physico-chemical characteristics of scaffolds are of high importance for the guiding of cell behavior. For cell-free based-tissue engineering approaches, the scaffold must induce cells to attach themselves to the biomaterial and migrate into the scaffold thickness. First of all, scaffold surface hydrophobicity is a key factor for cell adhesion. Indeed, cell adhesion molecules tend to absorb at higher levels onto hydrophobic surfaces. For instance, there was reported a maximal protein absorption and fibroblast adhesion towards biomaterials exhibiting water contact angles in the range 60°–80° [66]. Secondly, surface charge also impacts cell attachment, and anionic sulfated polysaccharides are known to influence cell adhesion, migration, proliferation and differentiation through their ability to bind proteins, such as growth factors, proteases, and chemokines [25,52,55]. Concerning surface roughness, it was found that the effect depends on the cell type. Fibroblasts adhere to various surface roughness, but exhibit different morphologies. Indeed, fibroblasts spread with a flattened shape onto smooth surfaces, while the morphology is affected by the surface grooves for rough surfaces [67]. Scaffolds with architecture at the microscopic level provide a framework for cell attachment and distribution in the 3D micro-environment, since sub-cellular structure controls cell-cell inter-relationships [66]. Finally, cells are sensitive to the matrix stiffness, and scaffolds mechanically compatible with the tissue to replace, are favored. 

Indeed, stress concentrations tend to increase at the tissue-scaffold interface when the biomaterial is too stiff, while a mechanical failure occurs for a too weak scaffold. In addition, cells routinely contract to pull on the biomaterial to which they are attached [68,69,70]. For instance, fibroblasts contract when they spread and elongate to migrate into a scaffold. It was demonstrated that fibroblasts are able to generate contractile forces up to the order of hundreds of nanonewtons [68,71]. As a consequence, the cellular response depends on the scaffold capacity to accommodate deformation, and biodegradable elastomeric scaffolds find applications in soft-tissue repair [65,69]. In our study, PEUUF scaffolds exhibited a water contact angle around 79°, a functionalized surface with anionic sulfated fucoidan, microscopic pores and a pore wall roughness. With regards to all these parameters, PEUUF scaffolds seem appropriate for fibroblast adhesion and infiltration. Moreover, PEUUF scaffolds exhibited a great elastomeric behavior with a short linear region (strain < 0.1), a prolonged elastic buckling plateau (strain from 0.1 to 0.5) and a post-complete densification domain at very large strains. Finally, PEUUF scaffold morphology recovered upon removal of the load (data not shown). Therefore, PEUUF scaffolds might accommodate large deformation by buckling, making them appropriate for soft-tissue engineering.

PEUUF scaffolds exhibited a highly interconnected porous structure (porosity of around 83%) with a multi-scale pore size from microscopic to macroscopic pores, with an average pore size of 500 μm. Literature reports scaffold elaboration with various pore sizes, ranging from 20 μm to 1500 μm, with a generally average pore size below 500 μm, but a high porosity, and a multi-scale pore size might be an advantage in promoting cell migration and further tissue ingrowth [66,69]. As a matter of fact, high interconnectivity and permeability (increasing with pore size) are crucial for the provision of oxygen and nutrients maintaining cell viability in the scaffold inner regions. Pore size and interconnectivity also influence the cell adhesion and migration rate. Indeed, small pores are essential for initial cell attachment, due to an increase in the specific surface area with decreasing pore size [72,73]. The optimal pore size for cell infiltration depends upon the cell type, and it was demonstrated that fibroblasts preferentially attach to scaffolds with pore sizes below 160 μm, and fibroblast migration decreases as pore size increases from 90 to 150 μm, due to a more available adhesive ligand density [66,69,70,74]. However when pores are too small, cell infiltration is limited at the scaffold outer surface restricting cell migration towards the scaffold center due to a limitation in the cell movement direction. The cellular aggregation at the scaffold edge limits nutrient and oxygen diffusion, as well as waste removal, resulting in necrotic cores inside the scaffold [69,70,72,73]. With larger pores above 300 μm and high interconnectivity, cell migration into the scaffold and migration speed are enhanced. Moreover, the cell spreading over the pore walls is more effective, and cells exhibit an elongated pattern that is known to be important for directed cell migration and persistent motion. Indeed, it was demonstrated that cell speed at pore wall junctions is lower than the speed along the pore walls regardless of the pore size. As a consequence, high interconnectivity and large pores enhance cell speed, since the time spent at pore wall junctions is decreased [69,73,75,76]. In addition, if cell speed decreases when cells travel on larger pores, cells travel further into the scaffold due to a less erratic and more directional movement. As a consequence, the importance of high specific surface area essential for cell attachment is overcome by the presence of larger pores that lead to a better cell infiltration and migration into scaffold. Finally, larger pores are also essential for scaffold vascularization and to provide boundaries for the tissue regrowth [73]. In our study, we found that the peculiar morphology of PEUUF scaffolds with high porosity and multi-scale pore size allowed cell recruitment and scaffold colonization. Indeed, when the PEUUF scaffold was laid over a fibroblast layer, cells were able to attach themselves to the biomaterial and migrate and proliferate into the scaffold. After 30 days of migration, it was evidenced that these fibroblasts had spread over the pore walls in an elongated pattern.

### 4.3. Ability of PRP-PEUUF Scaffolds to Promote Cell Recruitment and Proliferation

PRP contains more than 300 biologically-active molecules, including diffusible factors and macromolecules, regulating cell metabolisms or cell-extracellular matrix (ECM) interactions [8]. Therefore, PRP has been largely used to supplement cell culture media, or to stimulate and accelerate tissue regeneration [9,77,78]. Although neither a standard operating procedure nor any quality procedure are available, the efficiency of PRP in the field of tissue engineering has been shown despite of its heterogeneity. Promising results have been obtained with PRP-containing scaffolds, because PRP derivatives are capable of promoting cell adhesion, migration, proliferation, differentiation and of stimulating tissue growth [79,80,81,82,83,84]. The incorporation of PRP into scaffolds is an advantageous approach because it represents a simple, efficient and cost-effective method, allowing the immobilization of a number of highly concentrated bioactive factors, creating an optimized micro-environment and impacting on tissue regeneration. In most studies, PRP was immobilized within scaffolds in a dry state, or was directly used in hydrogel form [79,80,81,83,84,85].

In our study, PRP was immobilized in wetted scaffolds in the form of a fibrillar gel onto the pore walls and across pores, serving as a biological support for cell adhesion and scaffold colonization in a 3D spatial distribution layout. With the appropriate PRP concentration, the open porosity of the scaffold was maintained that is of high importance for the migration of cells, the diffusion of nutrients, and the overall bio-integration and tissue regeneration, when the scaffold is implanted in vivo. It was found that both PEUUF and PRP-PEUUF scaffolds allowed cell recruitment and subsequent migration. Nevertheless, PRP-PEUUF scaffolds exhibited an increase of the initial cell infiltration by comparison with PEUUF scaffolds. In addition, while the proliferation seemed to stop between 20 and 35 days in PEUUF scaffolds, continuous migration and proliferation were found into PRP-PEUUF ones. The immobilization of PRP enhanced the cell initial recruitment, migration, and proliferation, probably due to chemotactic factors and the adhesive protein-fibronectin contained in PRP [82]. Moreover while cells remained flattened on the pore walls of PEUUF scaffolds, layer of cells bridging pores were found within PRP-PEUUF ones. PRP-PEUUF scaffolds also exhibited an increase of the collagenous matrix deposit. In addition, we found that non-functionalized scaffolds synthesized in an absence of fucoidan and impregnated with PRP did not lead to the 3D arrangement of cells inside the scaffold porous structure and to the increase of collagen deposit, attesting the importance of the synergic effect of the presence of platelet-derived growth factors with LMW fucoidan to improve cell response [8,86]. Indeed, it was reported that sulfated glycosaminoglycans are capable of promoting a collagenous matrix, thus making and structuring matrix macromolecules, which are key events in soft-tissue repair [28,86,87,88,89,90]. Overall, the improved cell infiltration and more rapid colonization into PRP-PEUUF scaffolds, associated with a peculiar 3D cell distribution inside the scaffold and a higher collagen synthesis, suggested that PRP-PEUUF scaffolds might lead to some improved performance in vivo through potentiated regenerative properties. Finally, it has to be pointed out that the clinical PRP formulation used in our study was prepared from pools of platelets in order to achieve a maximal standardization and avoid the response heterogeneity from the PRP batch. The PRP adsorption was also realized after the scaffold sterilization, in order to avoid the loss and inactivation of growth factors due to high temperature. Therefore, this method of bio-activation is a simple and reproducible technique that can be easily reproduced by surgeons at the surgical block.

## 5. Conclusions

Our work brought out an easy and versatile way of developing bio-activated elastomeric poly(ester-urea-urethane) scaffolds. By combining the LMW fucoidan functionalization with PRP immobilization, we developed a scaffold that was capable of recruiting cells, acting as a 3D support for colonization, as well as mimicking the extracellular matrix to provide biological cues leading to cell response promotion. The scaffolds possessed properties suitable for soft-tissue regeneration, and will be investigated further in future with respect to in vivo animal testing to assess the interactions between PRP-PEUUF scaffolds and tissues, and therefore the in vivo performance.
